# pH-depended protein shell dis- and reassembly of ferritin nanoparticles revealed by atomic force microscopy

**DOI:** 10.1038/s41598-019-53943-3

**Published:** 2019-11-28

**Authors:** Lukas Stühn, Julia Auernhammer, Christian Dietz

**Affiliations:** Physics of Surfaces, Institute of Materials Science, Technische Universität Darmstadt, Alarich-Weiss-Str. 2, 64287 Darmstadt, Germany

**Keywords:** Nanoscale biophysics, Molecular capsules, Self-assembly, Applications of AFM, Nanoparticles

## Abstract

Ferritin, a protein that is present in the human body for a controlled iron storage and release, consists of a ferrihydrite core and a protein shell. Apoferritin, the empty shell of ferritin, can be modified to carry tailored properties exploitable for targeted and direct drug delivery. This protein shell has the ability to dis- and reassemble depending on the pH value of the liquid environment and can thus be filled with the desired substance. Here we observed the dis- and reassembly process of the protein shell of ferritin and apoferritin *in situ* and in real space using atomic force microscopy. Ferritin and apoferritin nanoparticles adsorbed on a mica substrate exhibited a change in their size by varying the pH value of the surrounding medium. Lowering the pH value of the solution led to a decrease in size of the nanoparticles whereas a successive increase of the pH value increased the particle size again. The pH dependent change in size could be related to the dis- and reassembling of the protein shell of ferritin and apoferritin. Supplementary imaging by bimodal magnetic force microscopy of ferritin molecules accomplished in air revealed a polygonal shape of the core and a three-fold symmetry of the protein shell providing valuable information about the substructure of the nanoparticles.

## Introduction

Ferritin is an iron-storage core-shell-protein present in almost all living organisms. Ferritin has an outer diameter of approximately 12 nm containing iron in the form of ferrihydrite in its approximately 8 nm large core. In the body of animals, ferritin is utilized to store, transport and release iron in a controlled manner for the oxygen transport, indispensable to life, and can be exploited for different medical^1,2^ and electronic applications^3^. Depending on the environment and the ferritin subtype, ferritin features a high thermal stability up to 77 °C^4^. As other proteins^5^, apoferritin, the empty protein shell of ferritin, can be used to transport various substances, such as drugs for medical applications^6–8^. The empty shell of ferritin can be used as a platform for the synthesis of nanoscale materials^9^. This protein shell is known to dis- and reassemble depending on the pH value of the surrounding medium^10^: the protein shell is stable in acidic environments down to pH values of 3.4, whereas the core is stable down to pH 2.1^11^. Below pH 3, the protein shell starts to disassemble. However, this process is reversible by the subsequent increase to higher pH values turning apoferritin to an almost ideal candidate as carrier for drug delivery, i.e. loading with a substance and subsequently release it. This loading occurs in a random fashion: the substance to be enclosed inside the protein shell is step-wise surrounded by the reassembling shell. It is possible to adsorb ferritin onto a substrate^12^ to study dis- and reassembling of the protein shell using sedimentation-velocity measurements^10^ and small angle x-ray scattering^11,13^. However, these studies do not provide a direct observation of the dis- and reassembling process and hence direct evidence, instead the conclusions had to be drawn based on subsequent transformation of the data. In particular for scattering methods, the observed data reflects the protein state in the reciprocal space and must be subsequently transferred to real space by means of simulations based on specific assumptions. Here, we report on the direct *in situ* observation of the pH dependent dis- and reassembly process of ferritin and apoferritin nanoparticles using atomic force microscopy (AFM) based on images that provide the size of individual particles in real space in liquid solutions of various pH values. Additionally, we examine the electrosteric interactions between the tip of an AFM cantilever, the ferritin nanoparticle and the substrate at different pH-values to validate the size interpretation of the molecules. Exploiting the coexistence of mechanical tip-shell forces and the magnetic tip-core interactions with bimodal magnetic force microscopy^14–16^ allowed us to resolve substructures of the molecules, such as the three-fold symmetry of the protein shell and a polygonal shape of the core indicating its crystalline condensed state.

## Results

### Electrosteric interactions

**Figure 1:**
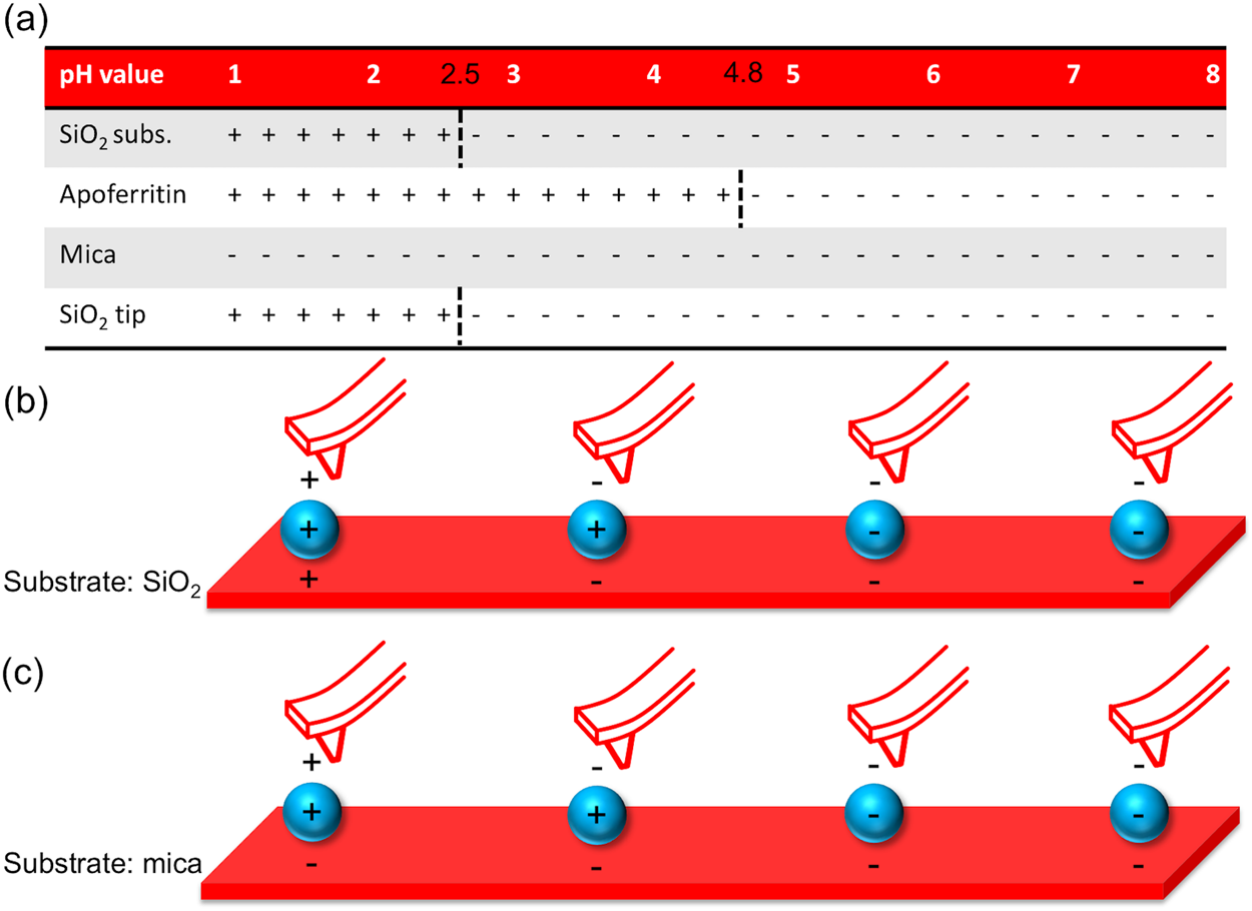
Surface charges of SiO_2_, mica and apoferritin nanoparticles at different pH values. (**a**) Net charge of the different surfaces. (**b**) Electrostatic interactions between an AFM tip, apoferritin and a SiO_2_ substrate or (**c**) muscovite mica in solutions of different pH values (pH 2, 4, 6 and 9), respectively.

The isoelectric point is the pH value of a liquid solution, at which the surface of an immersed material or molecule does not carry an electrical net charge. For proteins, this is also the pH value for best solubility. In Fig. 1a the sign of the surface net charge of silicon dioxide^17^, muscovite mica^18^ and apoferritin^19^ depending on the pH-value of the medium is depicted. The resulting charge interactions between the AFM tip, nanoparticle and SiO_2_ or mica for solutions in different pH-values are summarized in Fig. 1b,c, respectively.

As can be deduced from Fig. 1 attractive forces between the ferritin protein shell and the silicon substrate are only present in solution with pH values between approximately 2.5–4.8. For a mica substrate, attractive forces between ferritin nanoparticles and the substrate are also present for more acidic solutions. It can be concluded that a movement of the ferritin particles on a mica substrate by moderate tip-ferritin interactions is strongly inhibited in the above-mentioned pH ranges. The forces involved in the tip-ferritin interactions are net repulsive at pH values above 4.8 and below 2.5 and net attractive between 2.5 < pH < 4.8. The attractive interaction between the tip and the protein shell causes the *z*-piezo to move downward while scanning across an (apo)ferritin molecule whereas the actual size of the molecule forces the z-piezo to move up simply because of the topographical feedback that keeps the applied setpoint force constant. Thus, it is reasonable to believe that the height and hence the size of the ferritin nanoparticles at pH values in the range of 2.5 < pH < 4.8 is slightly underestimated by the apparent topography. If we detect a decrease in particle size in this range while lowering the pH value, then this decrease is an upper limit and might be even stronger in reality, compared to height changing values measured at pH < 2.5 or > 4.8 caused by the variation of the electrostatic interactions. This effect has to be taken into account when evaluating the development of the size of nanoparticles when changing the pH value of the solution.

### Dis- and reassembling of ferritin nanoparticles

**Figure 2:**
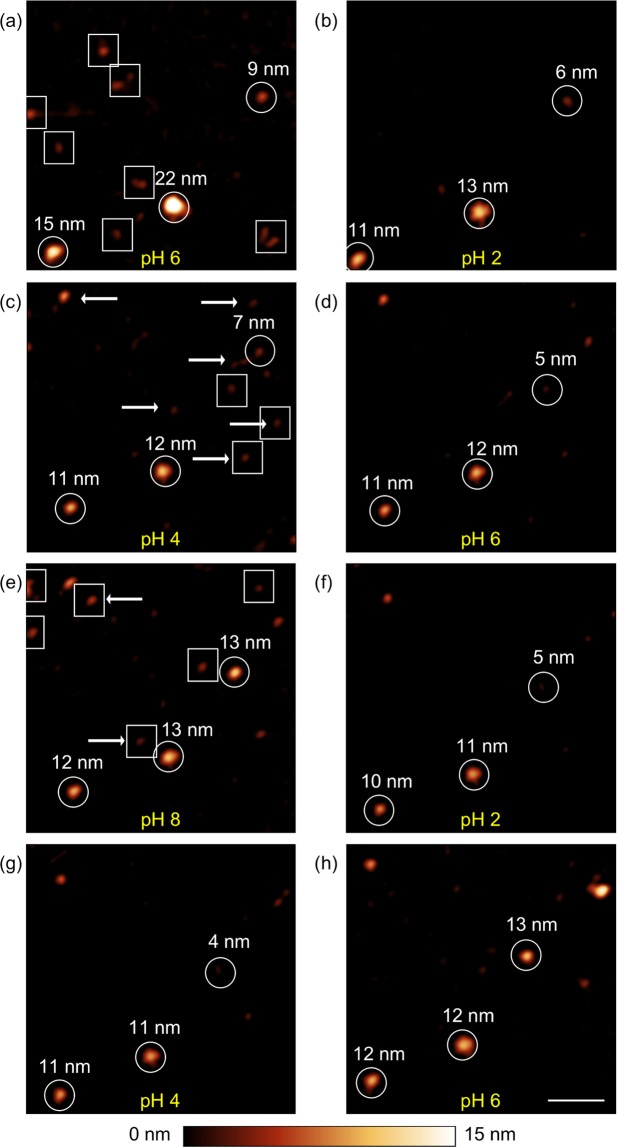
AFM topography images of ferritin nanoparticles adsorbed on a mica substrate measured in deionized water with adjusted pH value in the following order: (**a**) pH 6 (**b**) pH 2 (**c**) pH 4 (**d**) pH 6 (**e**) pH 8 (**f**) pH 2 **(g**) pH 4 (**h**) pH 6. Particles that vanished in the next step are enclosed by a square. Particles that assembled and did not exist in the previous step are marked by an arrow. Particles present through the whole experiment but vary in size are marked with a circle. Their sizes in nm are designated by numbers. Scale bar is 100 nm.

Figure 2 shows a series of topography images taken at the same spot of a mica substrate covered with ferritin nanoparticles. During the image acquisition, the pH value was varied in the following order: pH 6 (a), pH 2 (b), pH 4 (c), pH 6 (d), pH 8 (e), pH 2 (f), pH 4 (g), pH 6 (h). After each image acquisition, the adjusted pH value within the fluid cell was confirmed at the outlet of the cell and a new measurement at the next pH value was accomplished.

In Figure 2 a homogenous distribution of ferritin nanoparticles is apparent at the surface of the substrate. Individual particles could be identified, however their distribution in size was found to be polydisperse and the particles appeared slightly inhomogeneous in their shapes. In addition, laterally small and low objects in height assumed to be fragments of the protein shell were already found in the initial solution at rare locations. Particles that disappeared in the next step are enclosed by squares. We interpret the disappearance as complete dissembling of the protein shell accompanied by a release of the ferrihydrite core. Circles mark particles that are present throughout the entire measurement, yet varying in size (see number given above the particles in Fig. 2). Arrows indicate particles that were not apparent in the previous pH step and started to assemble. The maximum size of the particles changed during the course of the measurement series. The initial change from pH 6 (Fig. 2a) to pH 2 (Fig. 2b) caused a decrease in size of all nanoparticles marked with circles. The topmost particle among them changed its height from 9 nm to 6 nm, corresponding to the size of the ferrihydrite core. The particles marked with the squares are no longer visible in Fig. 2b. In the further course of the experiment when the pH value was step-by-step increased until pH 8 was adjusted (Fig. 2e), the size of the particles marked with the circles increased again, however they did not reach the initial value exactly, as measured in Fig. 2a. During the change of the pH value from pH 2 (Fig. 2b) to pH 8 (Fig. 2e), several new particles have formed as indicated by arrows. Similar phenomena were approved during a repeated pH change from pH 2 (Fig. 2e) to pH 6 (Fig. 2h). Summarizing the observations of the experiment: Decreasing the pH value of the solution causes a shrinkage of the nanoparticles in size whereas the subsequent increase of the pH value of the solution results in a growth of the particles and the formation of new particles.

### Dis- and reassembling of apoferritin nanoparticles

**Figure 3:**
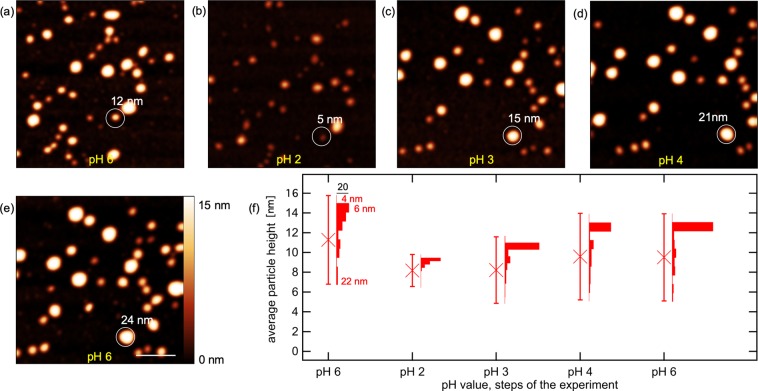
AFM topography images of apoferritin nanoparticles adsorbed on a mica substrate in solutions of different pH values: (**a**) pH 6 (**b**) pH 2 (**c**) pH 3 (**d**) pH 4 (**e**) pH 6. Scale bar is 100 nm. (**f**) Development of the average height (crosses) of the apoferritin nanoparticles adsorbed on a mica substrate in solution at different pH values analysed from the topography data. Error bars correspond to the standard deviation. Histograms show the height deviation at each pH value step.

To study the pH dependent size behaviour of apoferritin nanoparticles, topographical analysis by AFM in solutions of different pH values was performed in the same manner as for ferritin described in the previous section. The Apoferritin nanoparticles show a higher density on the substrate and a higher initial height deviation, than compared to the ferritin samples. The pH value of the solution was adjusted in the following order: pH 6, pH 2, pH 3, pH 4 und pH 6. Figure 3 shows the corresponding topography images of the measurement series. In contrast to the series accomplished on ferritin, the apoferritin sample exhibited a higher particle density on the surface for the prepared samples. The pattern of the nanoparticles and hence the particle distribution on the substrate partially changed with every step of varying pH value. This becomes particularly evident when the pH value of the solution was changed from pH 2 (Fig. 3b) to pH 3 (Fig. 3c). In addition to the increased number of nanoparticles on the surface compared to the experiment performed on the ferritin sample, the rearrangement of the particles was another reason why a statistical evaluation was preferred in that case. Hence, it was difficult to follow the presence of a particular nanoparticle during the course of the experiment.

A general change in size of the apoferritin nanoparticles by reducing the pH value is clearly apparent when comparing Fig. 3a,b: All apoferritin nanoparticles present exhibited a smaller size in topography in Fig. 3b compared to Fig. 3a, derivable from the generally darker colour value of the particles at the lower pH value. In the subsequent increase of the pH value from pH 3 to pH 6 (Fig. 3c–e), the initial size of the nanoparticles was restored. A statistical analysis of the size distribution of apoferritin at the different pH values based on the topography data is shown as histograms in Fig. 3f.

As for ferritin nanoparticles, it can be deduced that with decreasing pH value, the average height of the nanoparticles decreases, from approximately 11 nm at the first step of the measurement (pH 6) to approximately 8 nm in the second step (pH 2). The standard deviations of the size distribution displayed in Fig. 3f decreased too, from 10 nm at pH 6, to 7 nm at pH 2. The subsequent increase to pH 3 caused nearly no increase of the average height but an increase of the standard deviation from 4 nm to 8 nm. Further increase of the pH value led to an increase of the apoferritin nanoparticles in size to a value of 10 nm and standard deviation back to the initial values found at the beginning of the experiment at pH 6. We interpret the pH dependent decrease and increase of the ferritin and apoferritin molecules in size as pH-triggered dis- and resembling process of the protein shell.

### High resolution bimodal magnetic AFM measurements of ferritin

As demonstrated in the previous section, the pH dependent dis- and reassembling of ferritin and apoferritin particles can be directly visualized using atomic force microscopy. First high resolution studies on the protein shell of ferritin were performed by Ohnishi using contact mode AFM^20^. However, the core and shell substructure of ferritin molecules could not be resolved by this technique. In the following we show that by using bimodal magnetic force microscopy^15,16^, we can separate magnetic from mechanical cantilever responses of the mixed tip-sample interactions. This allowed us to image substructure features of the shell and the core of a single ferritin nanoparticle in great detail.

**Figure 4:**
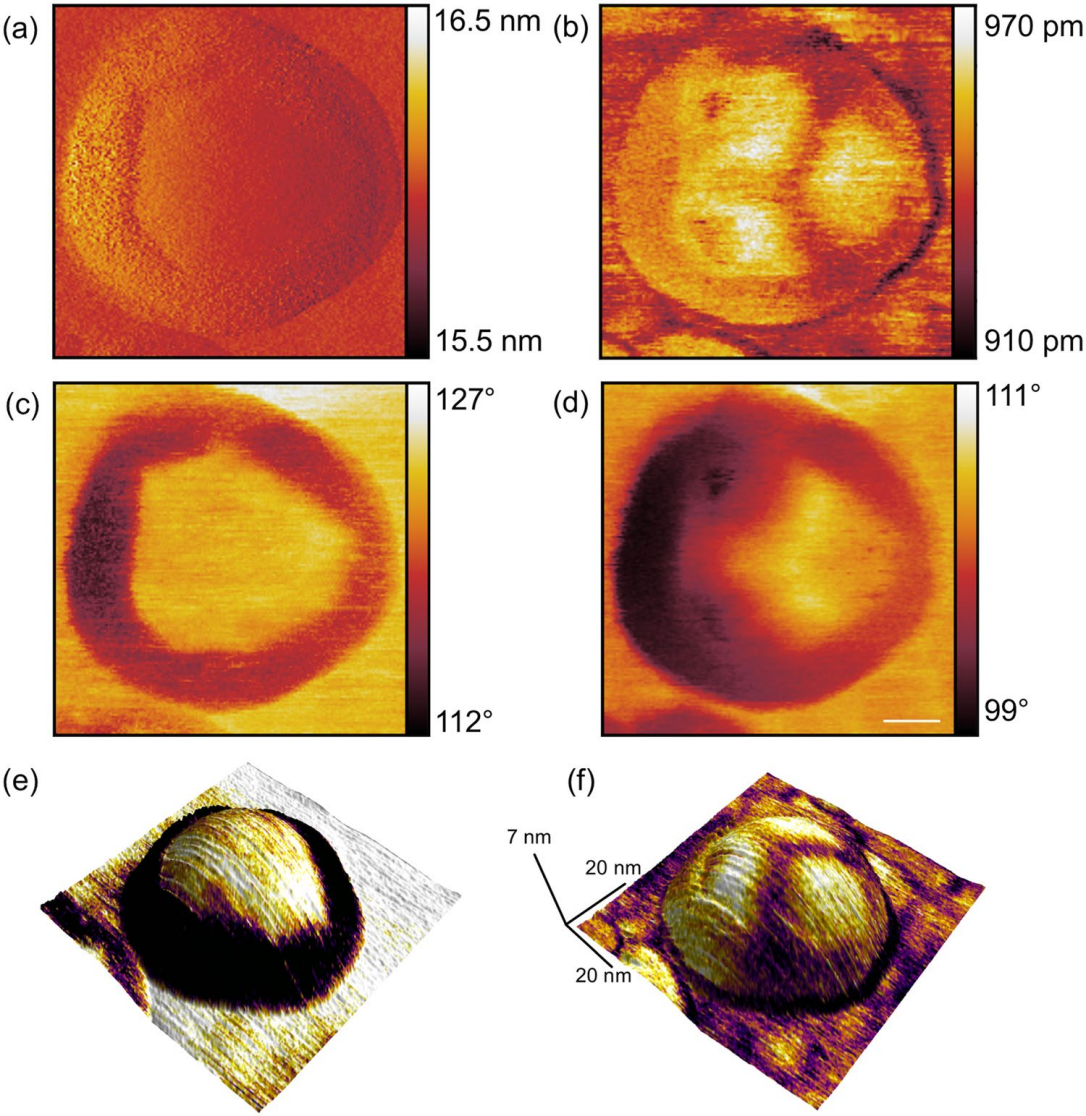
High-resolution bimodal MFM image of a single ferritin nanoparticle on a silicon substrate performed in air. (**a**) first and (**b**) second eigenmode amplitude, as well as (**c**) first and (**d**) second eigenmode phase images. Scale bar is 10 nm. (**e**) 3d topography with color-coded first eigenmode phase, (**f**) 3d topography with color-coded second eigenmode phase.

Figure 4 shows the five available observables of the bimodal MFM measurement, *i.e*. first (a) and second (b) eigenmode amplitude images as well as the first (c) and second (d) eigenmode phase images and a three-dimensional representation of the topography rendered by the colour code of the first and second eigenmode phase (Fig. 4e,f). Because of the use of a magnetic cantilever with a platinum/iridium coated tip exhibiting a larger tip radius as compared to standard cantilevers, the resulting diameter of the nanoparticle of almost 50 nm is significantly larger than its real diameter. The cores of the ferritin particles are superparamagnetic and can be polarized by the presence of an external magnetic field and/or the field originated by the oscillating magnetic tip. In the first eigenmode amplitude (Fig. 4a) and phase (Fig. 4c) images a ring around a centre area of constant amplitude and phase value, respectively, is apparent. In particular in the phase image of the first eigenmode it is obvious that this centre area is separated from the outer ring through linear edges intersecting each other by various angles forming a polygonal structure. Both second eigenmode observables (amplitude Fig. 4b, phase Fig. 4d) show a threefold symmetry in the centre area of the nanoparticle.

**Figure 5:**
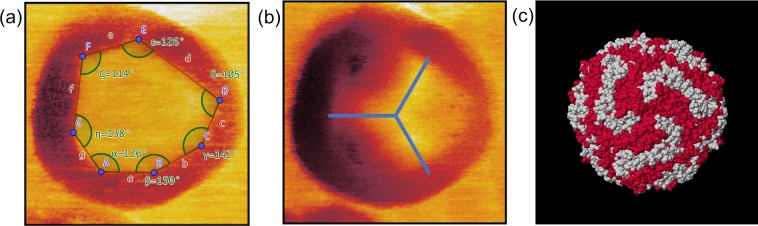
Analysis of the substructure features visible by bimodal MFM. (**a**) High-resolution first eigenmode phase image of a single ferritin nanoparticle on a silicon substrate performed in air. The separation lines between the outer ring and the inner central area as well as the corresponding angles at the intersections are highlighted. (**b**) Second eigenmode amplitude image exhibiting a threefold symmetry in the inner centre area. (**c**) Three-dimensional crystal structure of ferritin showing a threefold symmetry, calculated and created with Jmol^32^ (Jmol: an open-source Java viewer for chemical structures in 3D. http://www.jmol.org/, version 14) using data provided in refs. ^22,23^.

Further analysis of the polygonal structure is shown in Fig. 5a. The angles between the intersecting lines were measured to be α = 126°,β = 150°, *γ* = 142°, δ = 105°, ε = 126°,  ζ = 114° and η = 138°. Based on the variation of existing angles we conclude that the ferrihydrite core condenses in a complex morphologic state. The core can consist of different iron oxide phases (ferrihydrite, hematite, magnetite)^21^, which presents itself in our study as a (common) polygonal geometric structure. The analysis of the symmetry visible in the second eigenmode amplitude image was conducted in Fig. 5b. The bimodal excitation of the cantilever and the observation of the amplitude and phase shifts of the first two flexural eigenmodes in a single-pass technique allows for the simultaneous detection of mechanical and magnetic tip sample interactions at the nanometre scale^20^. By the choice of the experimental parameters (oscillation amplitudes *A*_1_, *A*_2_, amplitude ratio *A*_1_/*A*_2_, setpoint amplitude *A*_sp_/*A*_1_) it is determined whether the first or the second eigenmode channels are mainly dominated by magnetic or mechanical forces. Hence, the polygonal structure visible in Fig. 5a and the threefold symmetry in Fig. 5b is the result of a magnetomechanical contrast. This measured symmetry is in good agreement with the calculated structure visible in Fig. 5c. Figure 5c shows the symmetry of the protein shell of ferritin, calculated from X-ray data^22,23^. A threefold symmetry of the protein shell exposed to the topside, i.e. opposite to the substrate, is clearly visible in the image taken by MFM (Fig. 5b). Such symmetries arise at channels that form the protein shell^24,25^ based on the alignment of the protein molecules around the shell. In this study, we chose a relatively large first eigenmode amplitude enabling the first mode to be sensitive to magnetic interactions caused by the magnetic coating of the tip and the ferrihydrite core and a relatively high second eigenmode amplitude (compared to ref. ^20^) amplifying the mechanical contrast between the tip and the ferritin shell. For the measurement parameters used in this study such as free amplitude of the first eigenmode (*A*_1_ = 23 nm) and relative setpoint (*A*_SP_ = 16 nm) as well as the relatively large free amplitude of the second eigenmode (*A*_2_ = 16 nm) the observables of the first eigenmode are more prone for magnetic forces and the observables of the second mode are dominated by mechanical forces, which is in contrast to previous studies^19,20^ where substantially different parameters were applied, in particular a small second eigenmode amplitude. Thus, the first eigenmode (Figs. 4a,c and 5a) reveals the magnetic image of the particles generated by the ferritin core, the second eigenmode (Figs. 4b,d and 5b) the mechanical contrast given by the structure of the protein shell. A detailed study of the origin of the magnetomechanical contrast, however, is beyond the scope of this paper. Strikingly, as a result of this combination, a three-fold symmetry on the shell and the polycrystalline state of the core of this biomolecule became apparent in a single measurement.

## Conclusions

In conclusion, the pH dependent dis- and reassembly process of ferritin and apoferritin proteins can be directly visualized and studied in detail by an *in situ* experiment performed in an electrochemical cell of an atomic force microscope. The more acidic the environment of the molecules became, the lower was their measured size on average. The height of the molecules increased again when changing the solution to a neutral or basic milieu. A consideration of the surface charges of the AFM tip, protein shell and substrates allowed us to exclude artefacts in the observed trends arising from different electrostatic interactions. However, the attractive interaction in the range 2.5 < pH < 4.8 between tip and protein shell led to a generally reduced apparent height of ferritin and apoferritin molecules deduced from the topography images due to feedback restrictions. Thus, it is reasonable to believe that the real size of the molecules is slightly larger in that range. Strikingly, we observed an emergence/appearance of protein clusters at locations where no particle was present before. As the pH value was increased, new (apo)ferritin particles formed most likely developing from surrounding protein fragments, which remained in the liquid from previously disassembled protein shells. Due to the lack of a constant liquid flow in the electrochemistry cell, there is no reason to assume that the dissembled protein shell fragments were flushed away from the vicinity of the sample surface. Several authors^11,26^ assume that the higher-order structure of the protein shell is disassembled upon pH value decrease of the environment of the particles. However, the individual protein chains should remain intact. In the current study, conclusions about the structure of individual proteins cannot be drawn. Using atomic force microscopic measurements and further improving the resolution, it might pave the way to visualise the behaviour of individual proteins during the formation and degradation of the protein shell.

The bimodal excitation of a magnetic cantilever allowed us to simultaneously map the shape of a ferritin molecule, its mechanical as well as magnetic properties. In our experiments, these two properties could be clearly separated in the amplitude and phase channels of the different eigenmodes. The results suggest that the contrast found in the phase image of the first eigenmode mainly results from the magnetic interactions between the ferrihydrite core of the ferritin particle and the tip providing a defined polygonal structure as a result of the polycrystalline condensed state. Additionally, the contrast in the amplitude image deduced from the second eigenmode revealed a three-fold symmetry in the center of the ferritin nanoparticle arising from the mechanical interaction of the tip with the shell. This symmetry is in very good agreement with the calculated ferritin structure^22^ and the appearance demonstrates that the protein capsule was mainly intact.

In future, bimodal magnetic force microscopy in combination with a liquid environment of changeable pH value would allow for the observation of the dis- and reassembling process in great structural detail due to the simultaneous interplay of magnetic and mechanical forces between tip and sample. The methodology could open up new pathways to study drug release for targeted drug delivery in cancer research. However, magnetic force microscopy performed in liquids^20,27^ has been a great challenge so far due to the low-*Q* environment and unstable magnetic coatings, in particular on tiny magnetic structures such as present on nanoparticles.

## Experimental Section

### Sample preparation

To prepare ferritin samples, a two-step dilution of the stock solution was conducted. 10 µL of the purchased ferritin solution (10 mg/mL in 0.15 M NaCl) cationized from horse spleen (MFCD00081593, Merck KGaA, Darmstadt, Germany) were diluted into 1 mL deionized water (Milli-Q, Merck KGaA, Darmstadt, Germany) and homogenized for 5 min at 800 rpm at 22 °C (ThermoMixer, Eppendorf, Wessling-Berzdorf, Germany). 10 µL of the prepared solution were again diluted into 1 mL deionized water and homogenized repeating the procedure. 200 µL of the final solution were drop casted onto a freshly cleaved muscovite mica (50-D-10, NanoAndMore GmbH, Wetzlar, Germany) substrate or a 1 × 1 cm^2^ large piece of a Si(100) wafer (LamResearch Corporation, Fremont, CA). Size measurements with AFM were initiated 5 minutes after the samples preparation to provide sufficient time for the nanoparticles to settle on the substrate. For the apoferritin sample preparation, 10 µL of the apoferritin stock solution (MFC00081365, from equine spleen, 50% glycerol + 0.075 M NaCl), purchased from Merck KGaA as well, were drop casted onto the substrates (mica or Si(100)) and 200 µl deionized water were dropped above for further dilution. Size measurements by AFM were initiated after 5 minutes settling time. To observe the dis- and reassembly process of the molecules, particular attention was paid to avoid exsiccation of the sample after preparation to keep the tertiary structure of the protein shell intact.

### Size analysis by AFM

For the size analysis of the nanoparticles, PeakForce Tapping AFM measurements were carried out in a closed, heated electrochemistry cell (DIM EC cell, 932–012–300, Bruker, Santa Barbara, CA) mounted in an ICON AFM from the same manufacturer. Topographical images were taken using an oscillation amplitude of 300 nm at a driving frequency of 500 Hz, far less than resonance frequency of the cantilever in the solution to avoid resonance effects. The maximum force exerted to the sample was adjusted to 500 pN. The force applied is rather high and it can affect the analysis based on height measurements. Since the applied force was kept constant for all images such an effect applies to the whole imaging series and there is no change for the outcome of the experiment. Based on our observations we exclude particle migration or particle movement during the pH change because no traces that are typical for particle movement^28^ could be observed with the low force we adjusted. A structural change of e.g. the protein shell that would alter the observed height by changing the pH value seems unlikely to be the major origin of the size increase/decrease because of the relatively large size change we observe. The cantilevers used were SNL-A (Bruker AXS, Santa Barbara, CA), exhibiting a typical force constant of 0.35 N/m as determined by the thermal noise method^29^ and a nominal resonance frequency of 65 kHz in air.

Topography images were tilt and drift corrected by first order flattening. The temperature was set to 37 °C through the electrochemistry cell. The pH value of the measurement solution was adjusted to the desired values either by adding hydrochloric acid (Carl Roth GmbH + Co. KG, Karlsruhe, Germany) or ammonia (Carl Roth GmbH + Co. KG, Karlsruhe, Germany) to deionized water before filling the solution into a syringe. No additional ferritin or apoferritin nanoparticles are in the syringe-solutions. Using pre-heated (37 °C) syringes filled with the measuring solution of different pH values and a syringe pump Aladdin-1000 (World Precision Instruments, Sarasota, Florida), the pH value inside the electrochemistry cell was changed after each measuring step through connection tubes attached to the inlet of the cell. The pH stability of the solution in the cell during the measurement was confirmed at the outlet of the cell. The samples were kept in a liquid environment at all times. Due to the low number of particles, the size of the ferritin could be measured by drawing a cross-sectional profile through the particle and extracting the average height over a 3-pixel line with respect to the substrate level. The size of the apoferritin nanoparticles was analysed by an automated particle analysis tool^30^. Height profiles of individual particles can be found in the supplementary material (Figs. S1 and S3) and a trend of the change in height (Fig. S2).

### Substructure detection by bimodal MFM

To study the substructure of ferritin nanoparticles bimodal force microscopy^31^ was combined with magnetic force microscopy in a single-pass technique. Using a magnetic tip, it is feasible to separate long-range magnetic from short-range mechanical tip-sample interactions in air^19^ and in liquid environments^20^. To this end, we measured a ferritin sample in air using a Cypher S (Oxford Instruments, Asylum Research, Santa Barbara, CA) AFM with a magnetic Multi75-G cantilever (BudgetSensors, Sofia, Bulgaria) exhibiting a nominal force constant of 3 N/m and a nominal resonance frequency of 75 kHz of the fundamental flexural eigenmode in air. First and second cantilever eigenmodes were simultaneously excited by a photothermal excitation system and the respective amplitude and phase shifts of both modes were mapped. We used a scan speed of 1 Hz, free oscillation amplitudes of *A*_1_ = 23 nm and A_2_ = 16 nm and a setpoint amplitude ratio of *A*_sp_/*A*_1_ = 16 nm/23 nm = 70% to obtain images.

## Supplementary information

Supplementary dataset
