# Supplementary material for: pH-depended protein shell dis- and reassembly of ferritin nanoparticles revealed by atomic force microscopy
Source: Sci Rep. 2019 Nov 28;9:17755. doi: 10.1038/s41598-019-53943-3 (PMC6883049; doi:10.1038/s41598-019-53943-3)
Supplement: Supplementary file 1 — Supplementary dataset [file 41598_2019_53943_MOESM1_ESM.pdf]

pH-depended protein shell dis- and reassembly of ferritin nanoparticles revealed by atomic force microscopy

*Lukas Stühn[1], Julia Auernhammer[1], and Christian Dietz[1]**

[1] Physics of Surfaces, Institute of Materials Science, Technische Universität Darmstadt, Alarich-Weiss-Str. 2, 64287 Darmstadt, Germany

*corresponding author: dietz@pos.tu-darmstadt.de

Keywords: atomic force microscopy, bimodal magnetic force microscopy, ferritin, pH-dependent dis- and reassembly, magnetic nanoparticles

Supplementary Information

In addition to Figure 2, Figure S1 shows height profiles to the three nanoparticles marked with a circle in the manuscript figure.

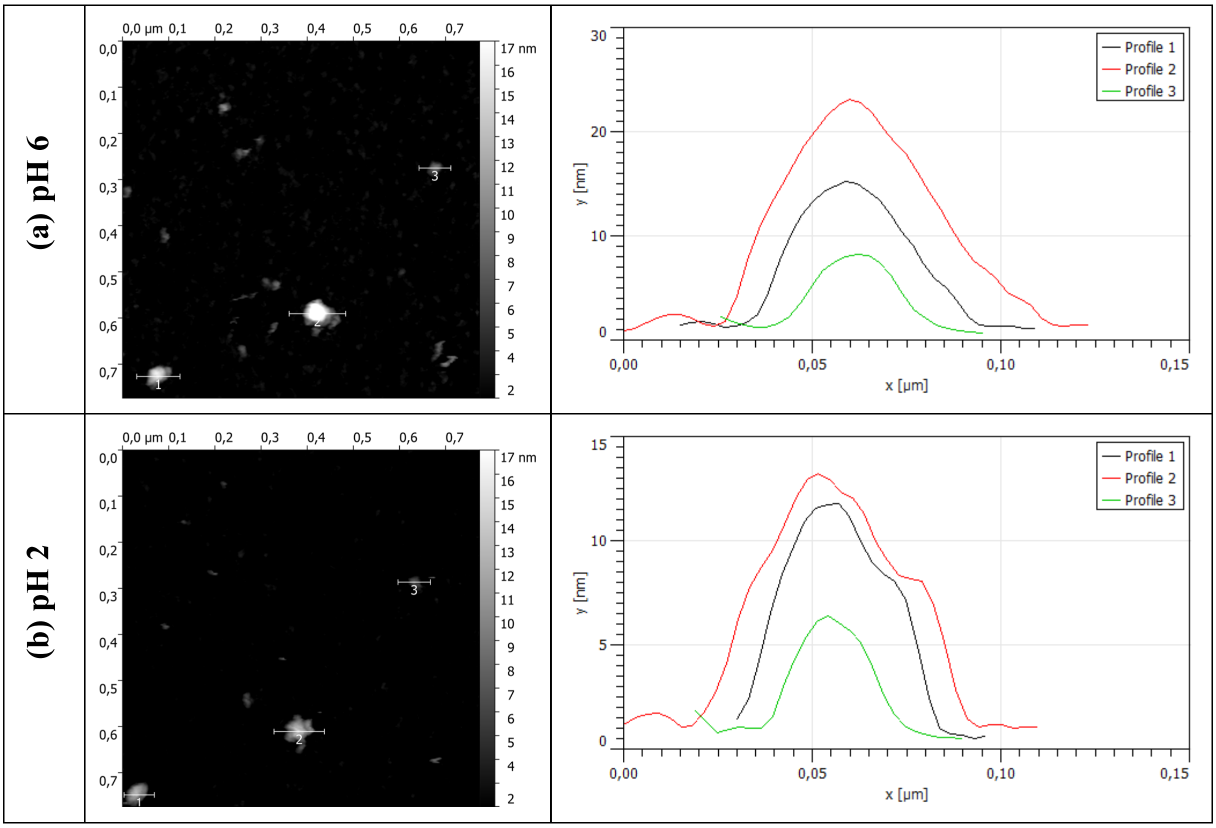

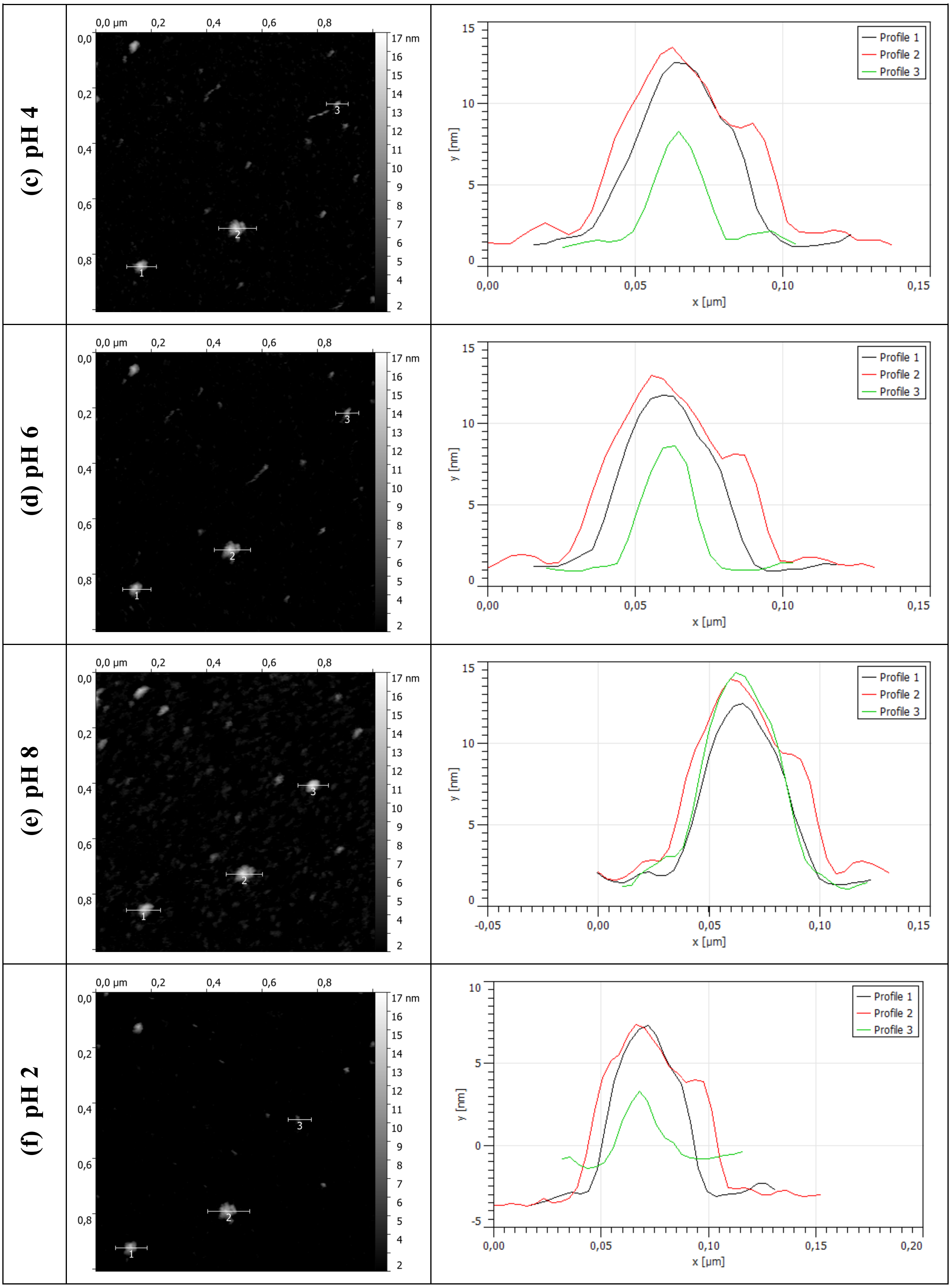

(c) pH 4
(d) pH 6
(e) pH 8
(f) pH 2
0,0 µm
0,2
0,4
0,6
0,8
17 nm
Profile 1
Profile 2
Profile 3
y [nm]
x [µm]

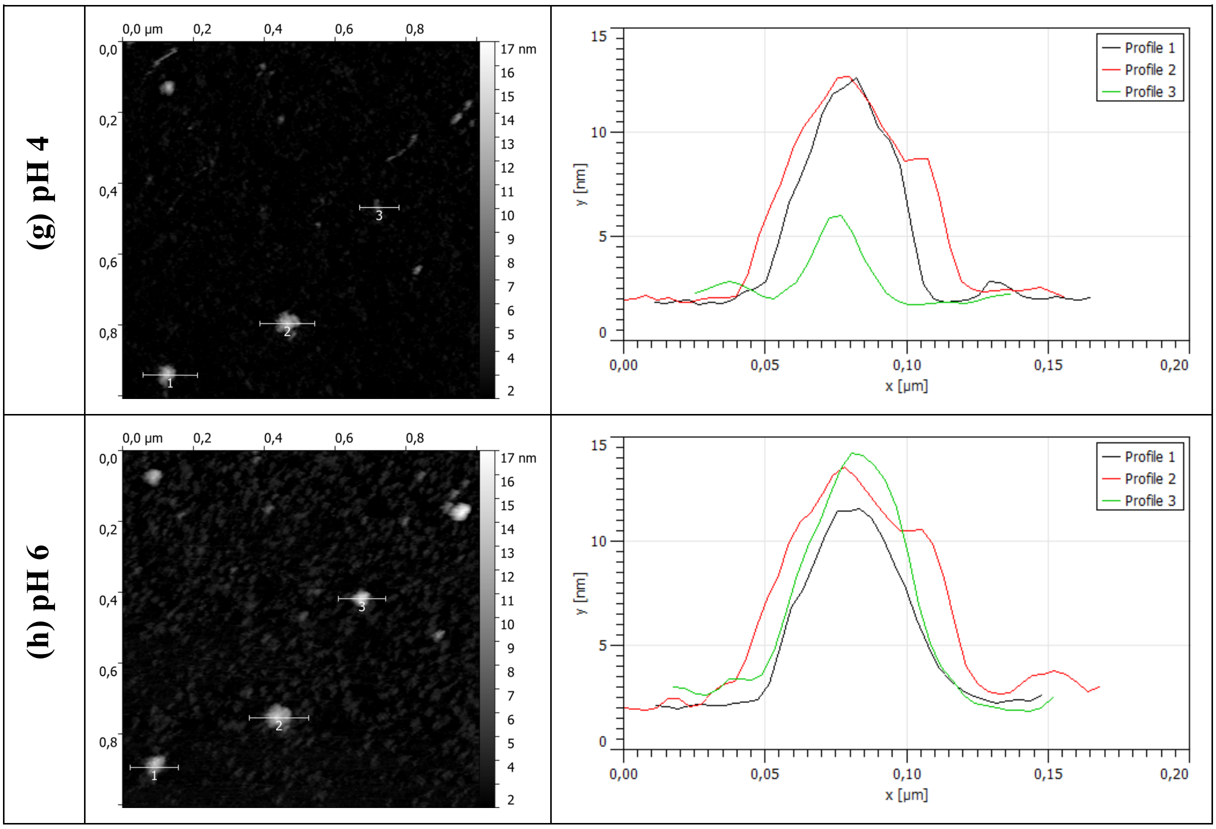

Figure S1 Height profiles corresponding to three individual ferritin nanoparticles at different pH value

Corresponding to the experiment shown in Figure 2, Figure S2 shows the changes in height of the three particles

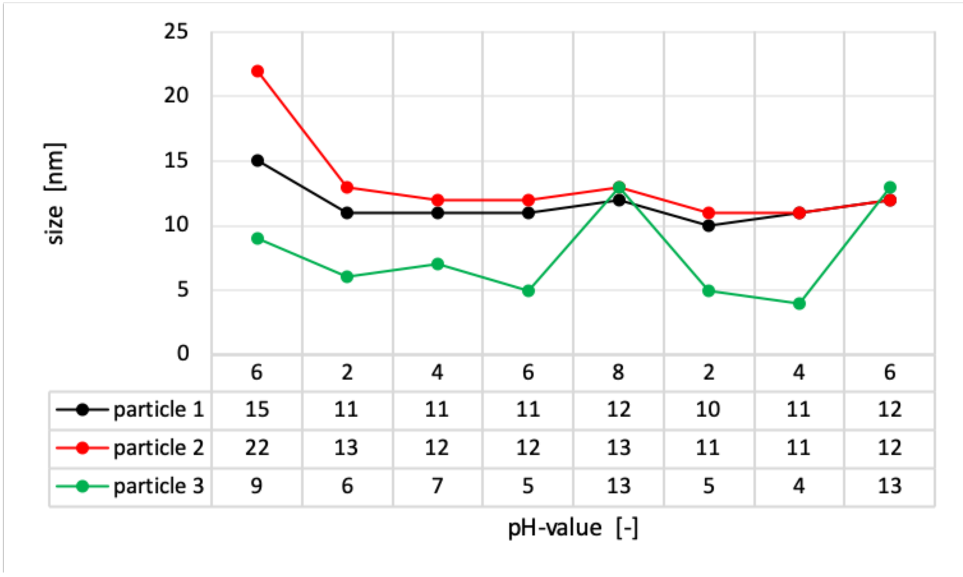

Figure S2 trend of height change during pH variation of three different ferritin nanoparticles

Corresponding to Figure 3, Figure S3 shows height profiles to 4 nanoparticles.

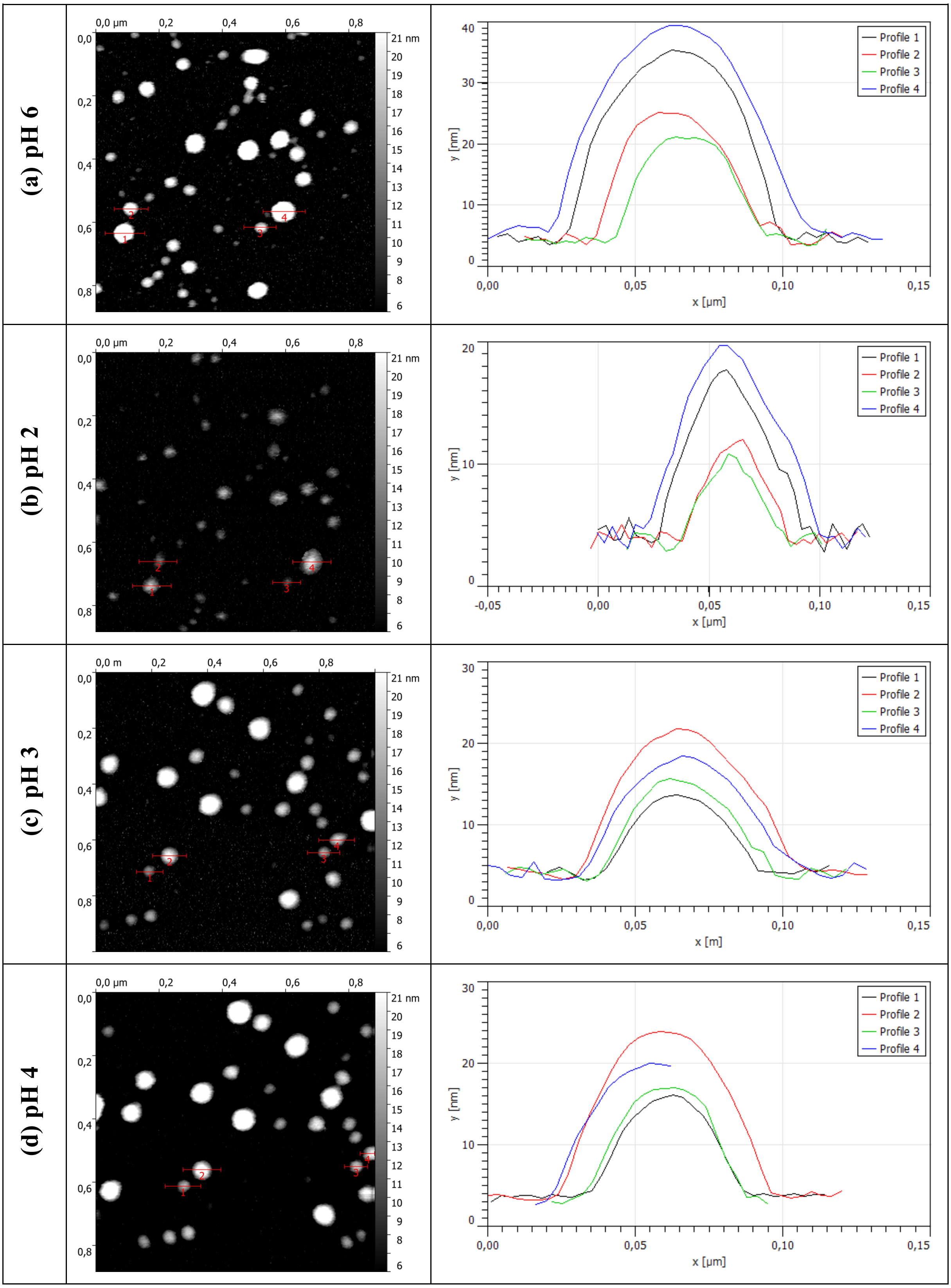

(e) pH 6

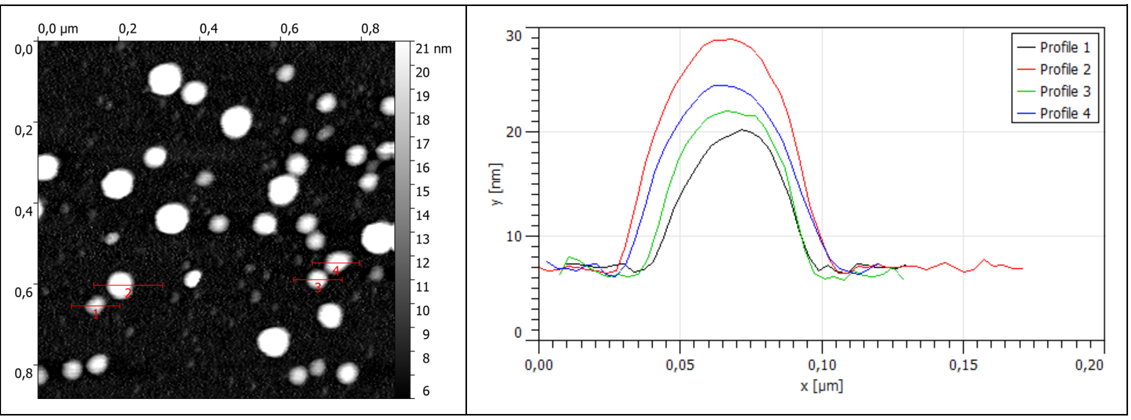

Figure S3 Height profiles corresponding to four individual apoferritin nanoparticles at different pH value
